# P-2144. Clinical Characteristics, Prognostic Factors, and Outcomes of Cytomegalovirus Infection in Intestinal Transplant Recipients Receiving Prophylaxis: A Single-Center Ten-Year Retrospective Cohort Study

**DOI:** 10.1093/ofid/ofaf695.2307

**Published:** 2026-01-11

**Authors:** Ming-Ying Ai, Wei-Lun Chang, Ming-Shyan Wang

**Affiliations:** Far-Eastern Memorial Hospital, New Taipei, Taipei, Taiwan (Republic of China); Far-Eastern Memorial Hospital, New Taipei, Taipei, Taiwan (Republic of China); Far-Eastern Memorial Hospital, New Taipei, Taipei, Taiwan (Republic of China)

## Abstract

**Background:**

High-risk intestinal transplant recipients (donor seropositive/recipient seronegative [D+/R-] or recipient seropositive [R+]) are recommended to receive CMV prophylaxis for 3–6 months. However, prior studies reported a high incidence of breakthrough CMV infections despite (val)ganciclovir prophylaxis, notably 95% (19/20 patients). D+/R- status remained an independent risk factor for CMV disease during prophylaxis. These patients require intensified immunosuppression, and valganciclovir absorption may be a problem. CMV disease is a major risk factor for graft rejection, emphasizing the need for improved prophylactic strategies. Our institution implemented a modified approach using renal-adjusted, treatment-dose (val)ganciclovir with close CMV viral load monitoring. This study describes CMV infection characteristics, prognostic factors, and outcomes under this strategy.

**Figure:**
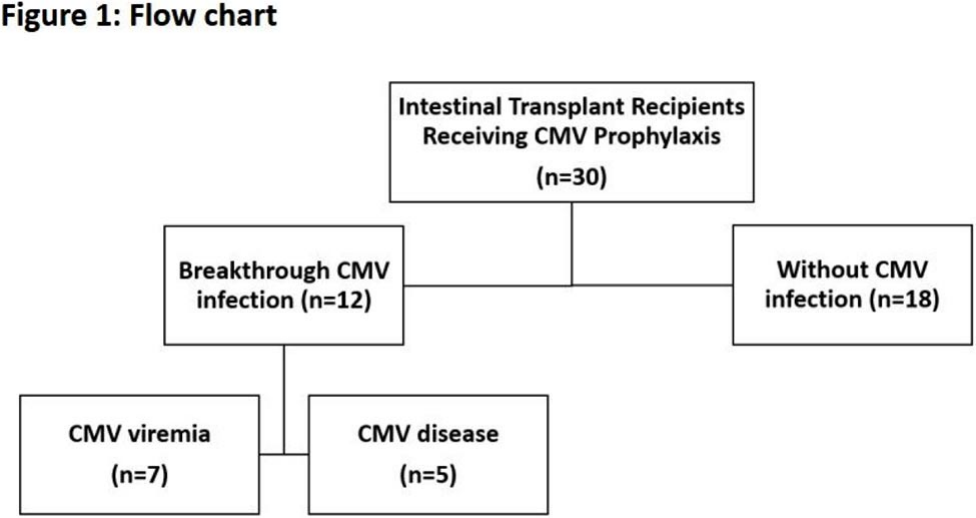

**Figure:**
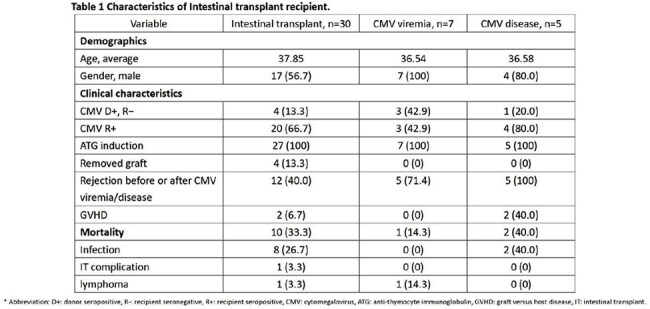

**Methods:**

This retrospective single-center study included intestinal transplant recipients from June 2014 to December 2024 who received renal-adjusted, treatment-dose (val)ganciclovir (5 mg/kg ganciclovir or 900 mg valganciclovir twice daily) for at least three months. Clinical features were described. Univariate analyses identified prognostic factors. Kaplan-Meier survival curves compared survival between recipients with and without CMV infection. A *p*-value ≤ 0.05 was considered significant.

**Figure:**
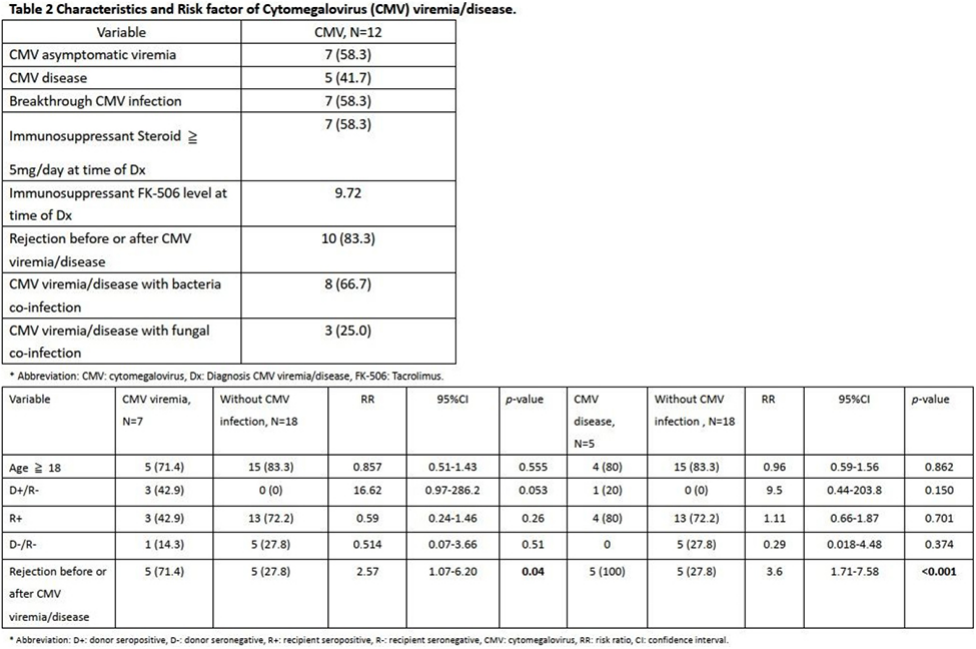

**Figure:**
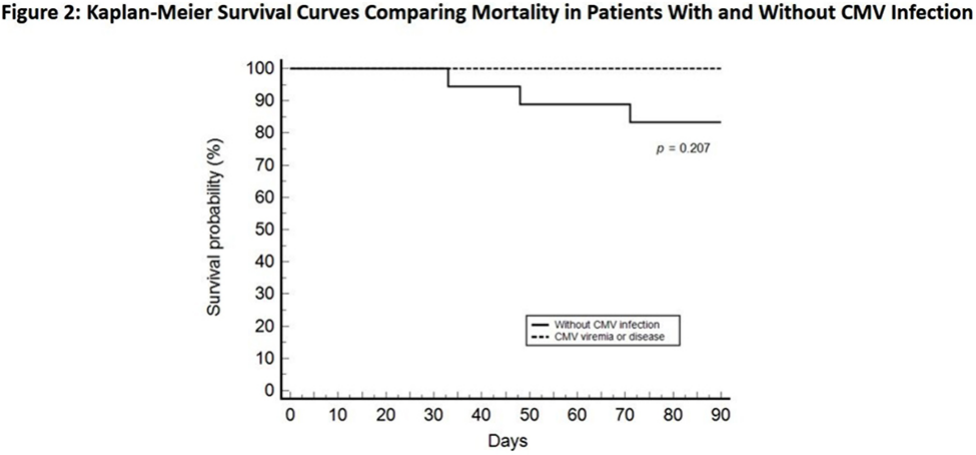

**Results:**

30 patients with a median age of 37.8 years were included; 13.3% were D+/R- and 66.7% were R+. CMV infection developed in 40% of recipients, including 7 asymptomatic viremia and 5 CMV disease cases. Notably, 16.7% developed CMV disease during prophylaxis. Rejection was a prognostic factor for CMV viremia (p = 0.040, RR 2.57) and disease (p < 0.001, RR 3.6). No significant difference in 90-day mortality was observed (p = 0.207). No adverse effects related to prophylaxis were documented.

**Conclusion:**

The modified CMV prophylaxis strategy substantially reduced breakthrough infections compared with previous reports and was well tolerated during prophylaxis. Rejection, rather than D+/R- status, was primary risk factor for CMV disease under this strategy. Combining intensified prophylaxis with close CMV monitoring may effectively prevent CMV disease in intestinal transplant recipients.

**Disclosures:**

All Authors: No reported disclosures

